# Comparative Metabolomics Unravel the Effect of Magnesium Oversupply on Tomato Fruit Quality and Associated Plant Metabolism

**DOI:** 10.3390/metabo9100231

**Published:** 2019-10-16

**Authors:** Min Cheol Kwon, Yangmin X. Kim, Seulbi Lee, Eun Sung Jung, Digar Singh, Jwakyung Sung, Choong Hwan Lee

**Affiliations:** 1 Department of Bioscience and Biotechnology, Konkuk University, Seoul 05029, Korea; alscjf1102@naver.com (M.C.K.); singhdigar@gmail.com (D.S.); 2 National Institutes of Agricultural Sciences, Rural Development Administration, Wanju 55365, Korea; yangmink@korea.kr (Y.X.K.); seulvi23@korea.kr (S.L.); 3 Department of Systems Biotechnology, Konkuk University, Seoul 05029, Korea; jes708@naver.com; 4 Department of Crop Science, College of Agriculture, Life and Environment Sciences, Chungbuk National University, Cheongju 28644, Korea; 5 Research Institute for Bioactive-Metabolome Network, Konkuk University, Seoul 05029, Korea

**Keywords:** tomato, magnesium oversupply, metabolomics, metabolic pathway, fruit quality

## Abstract

In general, greenhouse cultivation involves the rampant application of chemical fertilizers, with the aim of achieving high yields. Oversaturation with mineral nutrients that aid plant growth, development, and yield may lead to abiotic stress conditions. We explore the effects of excess magnesium on tomato plant metabolism, as well as tomato fruit quality using non-targeted mass spectrometry (MS)-based metabolomic approaches. Tomato plants were subjected to three different experiments, including high magnesium stress (MgH), extremely high magnesium stress (MgEH), and a control with optimal nutrient levels. Leaves, roots, and fruits were harvested at 16 weeks following the treatment. A metabolic pathway analysis showed that the metabolism induced by Mg oversupply was remarkably different between the leaf and root. Tomato plants allocated more resources to roots by upregulating carbohydrate and polyamine metabolism, while these pathways were downregulated in leaves. Mg oversupply affects the fruit metabolome in plants. In particular, the relative abundance of threonic acid, xylose, fucose, glucose, fumaric acid, malic acid, citric acid, oxoglutaric acid, threonine, glutamic acid, phenylalanine, and asparagine responsible for the flavor of tomato fruits was significantly decreased in the presence of Mg oversupply. Altogether, we concluded that Mg oversupply leads to drastically higher metabolite transport from sources (fully expanded leaves) to sinks (young leaves and roots), and thus, produces unfavorable outcomes in fruit quality and development.

## 1. Introduction

Tomato (*Solanum lycopersicum* L.) is the second most important vegetable crop in the world and attracts a great deal of attention, particularly in the face of increasing world populations and increasing demands [1]. Since the 1970s, there has been an exponential trend for the greenhouse cultivation of important vegetable crops, including tomatoes, under controlled micro-climate conditions that ensure their year-round supply [2]. Greenhouse cultivation raises land use rates, leading to intensive farming of crops. Furthermore, the greenhouse environment does not allow rain to reach the surface of the soil, and thus, repeated cultivation may result in the accumulation of certain nutrient salts (e.g., Mg^2+^, Ca^2+^, Na^+^, SO_4_^2−^, and Cl^−^) [2]. The excessive accumulation of nutrient salts imposes both ion toxicity and osmotic stress on plants and generates reactive oxygen species (ROS), which may lead to the oxidative destruction of plant cells [3]. Field-grown tomato plants are also cultivated using saline drainage water, and it contains not only high Na, but also high Mg [4].

Magnesium (Mg), one of the most abundant macronutrients in plants, mediates various physiological and biochemical processes, such as chlorophyll biosynthesis, enzyme activation, and carbon fixation by acting as the cofactor for a series of enzymes involved in these biosynthetic pathways [5,6]. Intriguingly, Mg is a phloem-mobile macronutrient and is essential for fruit maturation; however, its oversupply and deficiency may produce detrimental effects and result in abnormal plant growth [7]. Although Mg plays an important role in the growth and development of higher plants, it has attracted incidental attention from botanists and agronomists relative to other macronutrients and is often considered “the forgotten element” [8,9]. Additionally, Mg deficiency is known to diminish plant productivity and produce poor fruit quality [9,10]. However, the effects of Mg oversupply on fruit quality are not understood comprehensively. Therefore, it is important to delineate the molecular mechanisms associated with Mg oversupply and its effects on fruit development. Mg is involved in the export of carbohydrates from source to sink tissues [10]. Since Mg is a crucial component of many signaling and metabolic pathways, the effects of Mg imbalances on metabolic processes appear to be realized rapidly and differentiated between source and sink tissues [11]. Because of this, it is also important to examine the entire metabolisms of shoots and roots in order to evaluate the molecular mechanism of Mg oversupply on tomato fruits. 

Studying the plant metabolome and associated phenotypes provide a metabolic underpinning of plant development under varying abiotic stress conditions, its resilience functions, and the effects on crop quality, as well as yield [12,13]. Under abiotic stress conditions, plants display altered levels of a variety of biomolecules that affect their physiological adaptations and stress responses. Most notably, the primary metabolism, which is closely linked to normal growth and development in plants, plays an essential role in resource allocation, energy storage, and cell signaling mechanisms mediated by a plant’s gamut of metabolites [14]. In this study, we performed an untargeted metabolomic profiling assay using the high-throughput GC-TOF-MS and UHPLC-LTQ-Orbitrap-MS platforms to study the metabolic response of tomato leaves, roots, and fruits to Mg oversupply.

## 2. Results

### 2.1. Fruit Quality Traits and Bio-Activities Induced by Mg Oversupply

**Figure 1:**
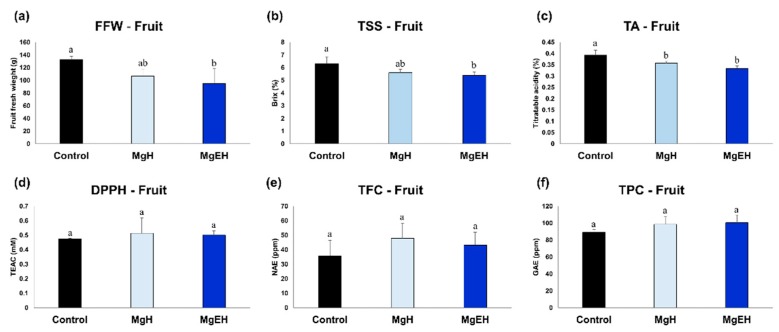
Effect of Mg oversupply on the fruit fresh weight (**a**), total soluble solid (**b**), and titratable acidity (**c**). Results of bioactivities [1,1-diphenyl-2-picrylhydrazyl (DPPH) (**d**), total flavonoids contents (TFC) (**e**), total phenolic contents (TPC) (**f**)] in tomato fruit induced by Mg oversupply. Different letters in the bar graph indicate significant difference by ANOVA followed by Duncan’s multiple-range test (*p*-value < 0.05).

Considering the soil electrical conductivities (EC) of lower than 3.0 dS m^−1^ for growing tomatos in a greenhouse [15], Mg oversupply levels were determined as MgH (higher than control, but lower than 3.0 dS m^−1^) and MgEH (higher than 3.0 dS m^−1^; see Materials and Methods). Comparisons of fruit fresh weight (FFW) and physicochemical characteristics among the tomato fruit samples were examined to evaluate the quality characteristics of fruits (Figure 1a–c). The fruit fresh weight (FFW), total soluble solid (TSS), and titratable acidity (TA) from the tomato juice samples displayed a decreasing trend with increasing Mg concentrations. In particular, these fruit quality traits were significantly decreased in tomato plants subjected to MgEH treatment. However, the 1,1-diphenyl-2-picrylhydrazyl (DPPH) activity, as well as total phenolic contents (TPC) and total flavonoids contents (TFC), in tomato fruit were marginally increased in the treated groups (Figure 1d–f).

### 2.2. Effect of Mg Oversupply on Tomato Plant Metabolism

**Figure 2:**
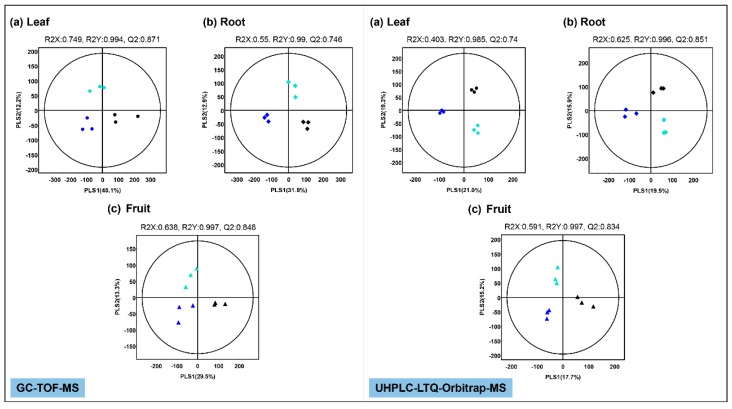
Partial least squares-discriminate analysis (PLS-DA) score plots for metabolites in tomato leaves, roots, and fruits under control and Mg oversupply conditions based on the GC-TOF-MS and UHPLC-Orbitrap-MS data set. (**a**) score plot for control (●), MgH (●), and MgEH (●) leaf samples, (**b**) score plot for control (♦), MgH (♦) and MgEH (♦) root samples, (**c**) score plot for control (▲), MgH (▲), and MgEH (▲) fruit samples.

To investigate the effect of Mg oversupply on tomato plant metabolism, we performed unsupervised PCA (Figure S1a,b) and supervised PLS-DA (Figure 2a,b) for tomato leaves and roots. Each multivariate statistical analysis consisted of three biological replicates per treatment. In leaves, the PLS-DA score plots based on GC-TOF-MS and UHPLC-LTQ-Orbitrap-MS exhibited (Figure 2a) a total variability of 57.3% (PLS1—45.1%, PLS2—12.2%) and 40.3% (PLS1—21.0%, PLS2—19.3%), respectively. In roots, two PLS-DA score plots exhibited (Figure 2b) a total variability of 44.7% (PLS1—31.8%, PLS2—12.9%) and 35.4% (PLS1—19.5%, PLS2—15.9%), respectively. The PLS-DA model validation was performed by determining the R2X, R2Y, and Q2 parameters. Based on the PLS-DA model for the respective profiling datasets, we selected significantly discriminant metabolites using variable importance in projection (VIP) values > 0.7. A total of 52 and 47 significantly discriminant metabolites were influenced following the Mg oversupply in leaves and root sample extracts, respectively, in tomato plants. 

The relative metabolite levels of tomato leaves and roots induced by Mg oversupply are shown in heat map (Figure S2). Among the primary metabolites (Figure S2a,b), we could confirm that the metabolite variations were marginal between MgH and MgEH treated samples; however, their relative abundances were considerably altered in MgEH group compared to the control groups. Interestingly, we could observe that metabolic variations of the above-ground (leaves) parts were different from the below-ground (roots) parts for both primary and secondary metabolites profiles. The relative levels of organic acids, carbohydrates, and polyamines were increased in the parts below-ground, but decreased in the parts above-ground, which suggests a mechanism behind their differential response to Mg oversupply. Furthermore, in tomato leaves, the relative levels of three polyamines and four phenylpropanoids were reduced following Mg oversupply; however, three steroidal saponins (hydroxytomatine, dehydrotomatine, and alpha-tomatine) were considerably higher compared to the control groups (Figure S2c).

**Figure 3:**
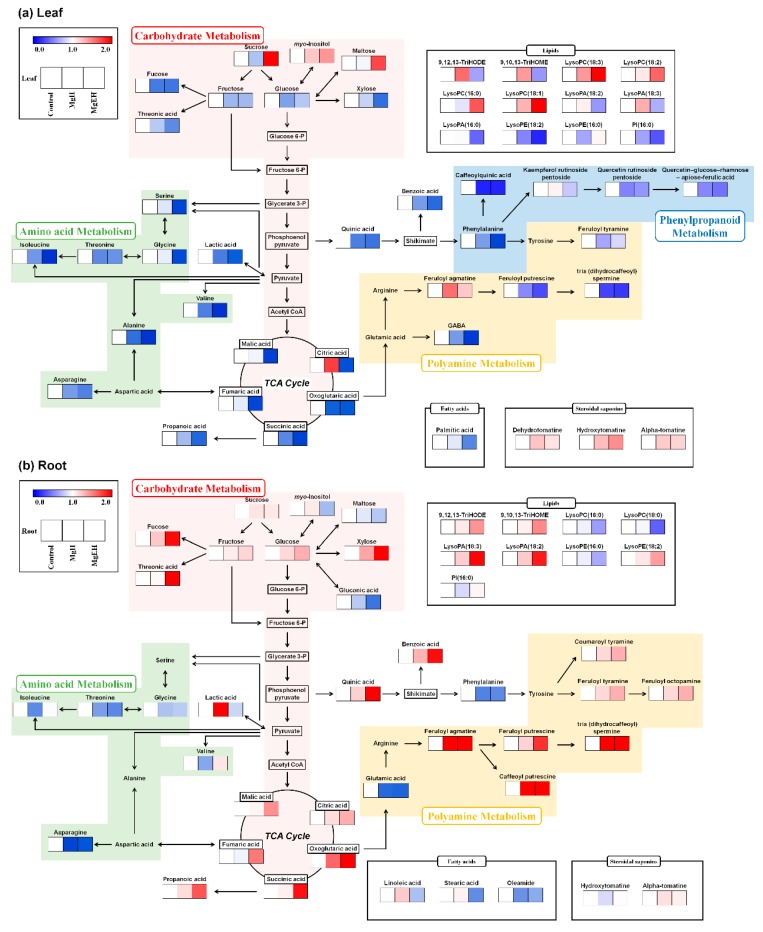
The constructed metabolic pathway for relative metabolite contents for tomato leaves (**a**) and roots (**b**) under Mg oversupply. The pathway was modified from the KEGG database (http://www.genome.jp/kegg/). The colored squares (blue-to-red) represent fold changes normalized by each metabolite level in the control group.

The relative contents of the discriminant metabolites among the tomato leaves and roots were comprehensively visualized in the corresponding metabolic pathway network layouts (Figure 3). Altogether, we observed the four different metabolic pathways, including carbohydrate, amino acid, polyamine, and phenylpropanoid metabolisms, were largely influenced by Mg oversupply. In roots, the carbohydrate metabolism linked to the TCA cycle appears to be upregulated, whereas, in leaves it appears to be downregulated. By contrast, the metabolic pathways related to amino acid biosynthesis were downregulated in both the leaf and root tissues under Mg oversupply conditions. However, the downregulation of amino acid metabolism was more severe in tomato leaves. The polyamine biosynthesis with precursors like glutamic acid and tyrosine displayed different patterns between tomato leaves and roots. In particular, the glutamic acid-derived polyamines (feruloyl agmatine, feruloyl putrescine, caffeoyl putrescine, and tris (dihydrocaffeoyl) spermine) and tyrosine derived polyamines (coumaroyl tyramine, feruloyl tyramine, and feruloyl octopamine derived from glutamic acid) were produced in higher volumes in the roots following Mg oversupply. On the contrary, most of the polyamines were decreased in leaves except for feruloyl agmatine. Additionally, phenylpropanoid metabolism associated with flavonoid biosynthesis was downregulated by Mg oversupply in leaf tissue.

### 2.3. Effect of Mg Oversupply on Tomato Fruit Metabolites

The unsupervised PCA (Figure S1c) and the supervised PLS-DA (Figure 2c) data indicated the marked effects of Mg oversupply on tomato fruit metabolite profiles. PLS-DA score plots based on GC-TOF-MS and UHPLC-LTQ-Orbitrap-MS datasets indicated clustered patterns between the control and Mg oversupply-treated groups (MgH and MgEH) along with total variability of 42.8% (PLS1—29.5%, PLS2—13.3%) and 32.9% (PLS1—17.7%, PLS2—15.2%), respectively. This stark separation within the dataset between samples from the control and treated groups indicated their distinct metabolite profiles. 

**Figure 4:**
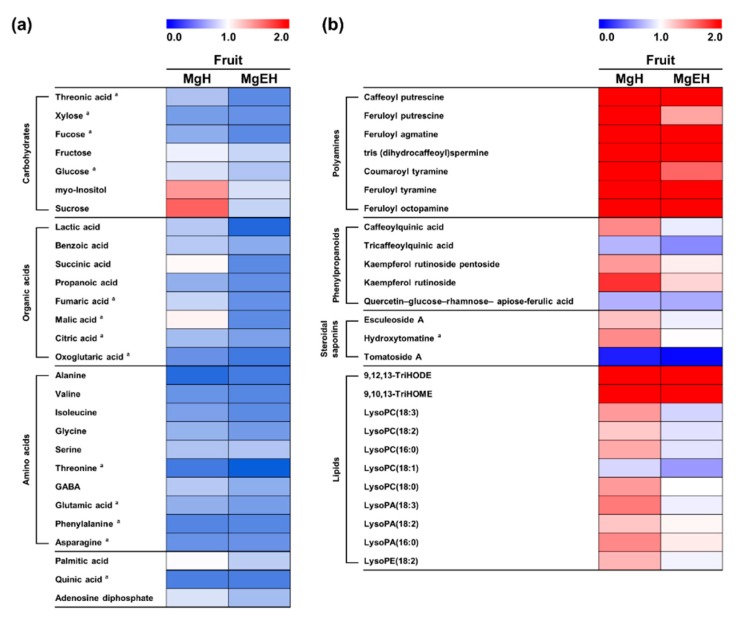
Heat map representation of the relative abundance of significantly discriminant fruit metabolites (VIP > 0.7) based on the fruit PLS-DA model (Figure 2c). The values represent the fold change with respect to control. ^a^ indicates significant differences (*p*-value < 0.05) between the control and each treatment. (**a**) Primary and (**b**) secondary metabolite data derived from GC-TOF-MS and UHPLC-LTQ-Orbitrap-MS, respectively.

The discriminant metabolites were selected based on VIP values > 0.7 of the tomato fruit PLS-DA model. A total of 54 significantly discriminant metabolites, including amino acids, organic acids, carbohydrates, fatty acids, polyamines, phenylpropanoids, steroidal saponins, and lipids were influenced by Mg oversupply (MgH and MgEH) and were selected from tomato fruit extracts (Tables S1 and S2). Notably, most of the primary metabolites (seven carbohydrates, eight organic acids, ten amino acids, and three miscellaneous ones) in tomato fruit extracts displayed lower abundance following the Mg oversupply treatments (Figure 4a). Among them, 13 of the primary metabolites (threonic acid, xylose, fucose, glucose, fumaric acid, malic acid, citiric acid, oxoglutaric acid, threonine, glutamic acid, phenylalanine, asparagine, and quinic acid) displayed statistically significant changes (*p*-value < 0.05) following the Mg oversupply treatments. Considering their effects on the fr’it's organoleptic traits, threonine and glucose mainly engender a sweet taste, while glutamic acid is highly correlated with an umami taste [16]. Furthermore, the phenylalanine contents influence the bitter taste, while citric acid and malic acid promote sourness in tomato fruits [16].

Considering the relative abundance of secondary metabolites (Figure 4b), altered levels of seven polyamines, five phenylpropanoids, three steroidal saponins, and eleven lipids were evident following the Mg oversupply. However, these changes were not statistically significant relative to their levels in the control groups. These results indicate that Mg oversupply may affect fruit metabolites, most notably the primary metabolites. The potential contribution of significantly discriminant metabolites to fruit quality traits was examined using statistical correlation analysis between their relative abundance and fruit quality traits, including FFW, TSS, and TA (Figure S3). Most of the primary metabolites with decreased abundance following the Mg oversupply showed a positive correlation with fruit quality traits. Among them, valine, isoleucine, glycine, threonine, glutamic acid, asparagine, and glucose displayed significantly positive correlations with TSS value, while alanine, valine, glycine, threonine, GABA, glutamic acid, phenylalaine, lactic acid, citric acid, oxoglutaric acid, threonic acid, and quinic acid showed significantly positive correlations with TA value (*p*-value < 0.05). This indicates that the reduction of primary metabolites by Mg oversupply is closely related to unfavorable fruit quality traits. On the contrary, secondary metabolites with altered abundance following Mg oversupply treatment displayed negative correlations with fruit quality traits.

## 3. Discussion

The metabolic changes induced by salinity stress in plants have been widely reported in the literature; however, our present study focused mainly on the metabolomic implications of Mg oversupply in tomato plants. We aimed to delineate the metabolomic effects of Mg oversupply on fruit quality, as well as the above/below-ground parts of a tomato plant. Considering the pl’nt's physiological response, the root is the first organ to encounter abiotic salt stress in the soil; this often limits the biomass production capability of plants [17]. Roots act as anchors for the plants and play a major role in plant nutrition and water uptake [18]. Intriguingly, the metabolic pathway mapping that associates the relative levels of discriminant metabolites unraveled the distinct metabolic patterns for above (leaves) and below (roots) ground plant parts (Figure 4). A variety of carbohydrates and organic acids related to glycolysis / TCA cycle were upregulated in tomato roots (Figure 3b). In response to salt stress, carbohydrate accumulation in plant roots maintains iso-osmotic conditions and promotes rapid cell wall synthesis [19], fulfilling the heightened energy demands. Additionally, some carbohydrates function as signaling molecules when plants are exposed to salt stress [20]. Mg oversupply in our study would have resulted in 0.5–0.7 MPa osmotic pressure, which is comparable to root cell turgor pressure [21] if the exchangeable Mg in the soil was dissolved in a soil solution at pH 7. The osmotic pressure in the soil may be even higher when considering the excess of anions. This would have caused osmotic stress to tomato roots without osmoregulation. When tomato roots were exposed to Mg oversupply, the soluble sugars like glucose, sucrose, fructose, and fucose were significantly accumulated compared to the control (Figure S2b). Our results are congruent with the study published by Peng et al. which reported that soybean roots exhibit a higher abundance of sucrose and starch under high Mg conditions [22]. Carbohydrate accumulation in the roots and stress responses under Mg oversupply conditions can be hypothesized based on the source-sink balance perspective. Carbon allocation patterns are very complex in plants, owing to the multiple-age structures of roots and leaves [23]. According to previous reports, carbon allocation to the sink tissue is regulated by both the source and the sink [24]. Among the macronutrients required by plants, Mg plays an important role in phloem loading and the transport of photo-assimilates into sink organs, such as roots [25]. Thus, Mg oversupply in root zones would increase the carbohydrate demand, suggesting that carbohydrates travel from source to sink. Similarly, various sugars and organic acids related to carbohydrate metabolism might be down-regulated in tomato leaves. According to previous reports, starch and sucrose were significantly decreased in leaves, but increased in roots when the Mg supply was increased [22]. These soluble sugars play essential roles in carbon storage, osmo-protection, and the scavenging of free radicals [26].

Significant reductions in TCA cycle intermediate contents in leaves (Figure 3a) coupled with increases in these organic acids in the roots (Figure 3b) under Mg oversupply conditions are suggestive of higher biosynthesis of TCA cycle intermediates. This would imply that different plant organs (leaves and roots) have different metabolic activities under Mg oversupply, which accentuates the importance of root metabolic activities. In fact, the TCA cycle, which is an important energy-producing process in plants, plays an important role in resisting adverse environmental conditions [27]. The TCA cycle intermediates accumulated substantially in the roots under Mg oversupply. Therefore, the TCA cycle can enhance a tomato plant’s resistance to Mg oversupply by increasing the levels of TCA intermediate metabolites to meet the heightened energy demands, as reported previously for soybean roots [27].

Understanding amino acid metabolism and the regulation of polyamines in plants is a major goal because the metabolites of these two groups play important roles in linking several pathways involved in C, N, and secondary metabolism [28]. In this respect, glutamate is the key metabolite of C, N, and secondary metabolism because it can be converted into ornithine, arginine, GABA, and polyamines. In addition to the production of amino acids and signal molecules like GABA, this group of sub-pathways is the primary source of putrescine biosynthesis, which in turn produces the other two common polyamines, spermine and spermidine [28]. Opposite phenomena were observed in leaves and roots in polyamine metabolism from glutamic acid and phenylalanine (Figure 3a,b). Under various abiotic stresses, it has been reported that the level of polyamines increases strongly in plants [29]. However, Mg oversupply reduced polyamine levels in leaves, but increased their levels in roots. In general, salt stress in the soil increases free radical production in plant roots, thereby inducing polyamine biosynthesis [30]. Therefore, the accumulation of excess Mg in roots increased polyamine synthesis and decreased its precursor, glutamic acid (Figure 3b). We suggest that glutamic acid, a direct precursor of N-rich metabolites (polyamines), plays an important role in the Mg oversupply response in tomato roots. We observed that glycine, serine, GABA, and asparagine were significantly decreased in tomato leaves (Figure 3a and Figure S2b). Previously, it has been reported that the amino acid contents of tea leaves gradually decreased under high concentrations of Mg [31]. Functionally, Mg is vital for chlorophyll biosynthesis, and thus, photosynthesis; hence, both its oversupply and deficiency can harm plant growth and metabolism [7].

Given the comprehensive data that has been collected, our study demonstrated that Mg oversupply affected tomato fruit production and quality by altering their metabolic processes, although these effects may be depends on cultivars. Soil salinity is known to induce root apoplastic barrier and to increase the root biomass to provide the salt stress tolerance of citrus rootstock [32], and this is in line with higher resource allocation to tomato roots rather than fruits of our study. As shown in the heat map representation of the relative metabolite levels in tomato fruits (Figure 4a), Mg oversupply significantly reduces the levels of carbohydrates, organic acids, and amino acids and results in impaired fruit quality and organoleptic traits. The reduction of primary metabolites suggested that fruits would not have received adequate resource allocation because magnesium oversupply concentrated resource allocation from leaves to roots. Tomato taste depends on optimal concentrations of carbohydrates, organic acids, phenols, and mineral components with carbohydrates making the largest quantitative contribution [33]. We observed that the relative abundance of threonic acid, xylose, fucose, and glucose decreased significantly in the presence of Mg oversupply. Among them, the proportions of soluble sugars (glucose and fructose) are critical in regulating the sweetness and taste characteristics of tomatoes. Thus, it can be conjectured that Mg oversupply negatively affects important organoleptic traits, including sweetness and TSS in tomato fruits. The organic acids contribute to the sour taste in fruits imparting the desirable and balanced sweet-sour flavor in tomatoes. However, the relative abundances of organic acids (fumaric acid, malic acid, citric acid, and oxoglutaric acid) produced through the TCA cycle were significantly decreased after exposure to Mg oversupply (Figure 4a), which may have influenced the TA in tomatoes (Figure 1c). Reportedly, citric acid and malic acid are the most abundant organic acids in tomato fruits and determine their commercial organoleptic traits [34,35]. Considering the importance of the free amino acid composition of tomato fruits, the significantly reduced proportions of threonine, glutamic acid, phenylalanine, and asparagine following Mg oversupply may have affected associated flavors in tomatoes (Figure 4a). Notably, the four amino acids, including glutamic acids, GABA, aspartic acid, and glutamine, account for 80% of the total free amino acids in the tomato fruits [36]. Glutamic acids are known to provide the characteristic “umami taste” in fruits [37], while GABA synthesized from glutamic acid plays a major role in nitrogen and carbon metabolism, and is also an integral part of the TCA cycle under stress conditions [38]. Hence, the reduced biosynthesis of these amino acids in Mg oversupply-treated tomato plant groups can be considered detrimental to fruit flavor. In our correlation analysis (Figure S3), most of the primary metabolites were positively correlated with FFW, TSS, and TA. On the contrary, most of the secondary metabolites were negatively correlated with fruit quality traits. Hence, after careful analysis, we suggest that Mg oversupply has a large impact on the levels of primary metabolites that have downstream implications on the associated organoleptic traits responsible for determining fruit quality.

## 4. Materials and Methods

### 4.1. Chemicals and Reagents

Analytical grade methanol, acetonitrile, and water were purchased from Fisher Scientific (Pittsburgh, PA, USA). The reagent grade chemicals, including methoxyamine hydrochloride, pyridine, and N-methyl-N-(trimethylsilyl)-trifluoroacetamide (MSTFA), were obtained from Sigma Chemical Co. (St. Louis, MO, USA). 

### 4.2. Plant growth, Fruit Harvest, and Preparation

Three-month-old tomato seedlings (Solanum lycopersicum L., cv. Super Dotaerang; indeterminate type, fresh market variety, globe, hybrid; Koregon Co., Ltd, Anseong, Korea) were transplanted into 15 L plastic pots containing soil on April 5, 2018, and grown for another 16 weeks. The temperature was maintained between 15 °C and 35 °C with a daily fluctuation in a greenhouse in the National Institute of Agricultural Sciences, Rural Development Administration, in Jeonju, Korea. To investigate the effects of high magnesium supply, the initial fertilizer supply to the soil before transplanting and additional fertilizer supplies after transplanting contained 7.5 (MgH) or 10 (MgEH) times of the control level of magnesium. At the final harvest, the soil magnesium levels were at 1.3, 3.7, and 4.6 cmol kg^−1^ for the control, MgH, and MgEH groups, respectively. The electrical conductivities of soil for the control, MgH, and MgEH groups were 0.7, 2.5, and 3.4 dS m^−1^, respectively. The plants were provided with tap water daily. For the improvement of the fruit set, hormones (gibberellin and 4-chlorophenoxyacetic acid) were applied to fully expanded flowers. Ripened tomato fruits from 4 different plants in each treatment group were harvested at similar ripening stages that were judged by their color. At 10:00 a.m., the fruits were harvested in order to get rid of the diurnal fluctuations in metabolite contents that may exist. At the final harvest of fruits, there was a harvest of roots and leaves near the harvested tomato fruits (9–10th nodes from the bottom). Harvested leaves, roots, and fruits were washed with distilled water and water was removed before storing at −80 °C. Each sample was lyophilized for four days and then ground into a fine powder with a mortar and pestle. Each powdered sample was stored at −80 °C until metabolite extraction.

### 4.3. Sample Extraction 

Each dried powdered sample (100 mg) was extracted with 1 mL of 80% aqueous methanol using a MM400 mixer mill (Retsch^®^; Haan, Germany) at a frequency of 30 s^−1^ for 10 min, followed by 5 min of sonication at 4 °C (Hettich Zentrifugen Universal 320, Tuttlingen, Germany). Subsequently, the extracts were centrifuged at 13,000 rpm for 10 min at 4 °C, and the supernatants were filtered through 0.2 μm polytetrafluoroethylene syringe filters. The filtered supernatants were completely dried using a speed-vacuum concentrator (Biotron, Seoul, Korea). The dried samples were reconstituted with 80% aqueous methanol to a final concentration of 20,000 ppm (20 mg/mL) to be used for bio-activity assays and instrument analysis.

### 4.4. GC-TOF-MS Analysis

The dried samples were subjected to two steps of a derivatization reaction prior to GC-TOF-MS analysis. First, the oximation was performed by adding 50 μL of methoxyamine hydrochloride in pyridine (20 mg/mL) to the dried samples, and the reaction mixture was incubated at 30 °C for 90 min. Next, the silylation was performed by adding 50 μL of MSTFA to the reaction mixture, followed by a 37 °C incubation for 30 min. The final concentration of the derivatized samples was set at 20,000 ppm (20 mg/mL) with daidzein (0.25 mg/mL) as the added internal standard (IS). All the samples were filtered using a Millex GP 0.22 µm filter (Merck Millipore, Billerica, MA, USA) prior to instrument analysis. 

GC-TOF-MS analysis was performed using an Agilent 7890A GC system (Agilent Technologies, Palo Alto, CA, USA) with an Agilent 7693 autosampler and a Pegasus HT TOF-MS (Leco Corporation, St. Joseph, MI, USA). The analytical program employed for sample analysis was adopted from a previous study [39]. We maintained three biological replicates for each of the sample and the analyses were performed in random order to reduce the bias.

### 4.5. UHPLC-LTQ-Orbitrap-MS

A UHPLC system equipped with a Vanquish binary pump H system (Thermo Fisher Scientific, Waltham, MA, USA) coupled with auto-sampler and column compartment. Chromatographic separation was performed on a Phenomenex KINETEX^®^ C18 column (100 mm × 2.1 mm, 1.7 μm particle size; Torrance, CA, USA) and the injection volume was 5 μL. The column temperature was set to 40 °C, and the flow rate was 0.3 mL/min. The mobile phase consisted of 0.1% *v/v* formic acid in water (A) and 0.1% *v/v* formic acid in acetonitrile (B). The gradient parameters were set as follows: Five percent solvent B was maintained initially for 1 min, followed by a linear increase to 100% solvent B over 9 min and then sustained at 100% solvent B for 1 min, with a gradual decrease to 5% solvent B over 3 min. The total run time was 14 min. The MS data were collected in the range of 100–1500 *m*/*z* (under negative- and positive-ion mode) using an Orbitrap Velos ProTM system, which is combined with an ion trap mass spectrometer (Thermo Fisher Scientific, Waltham, MA, USA) coupled with a HESI-II probe. The probe heater and capillary temperatures were set to 300 °C and 350 °C, respectively. The capillary voltage was set to 2.5 kV in negative mode (positive mode, 3.7 Kv).

### 4.6. Determination of Fruit Quality Traits (Total Soluble Solid Contents and Titratable Acidity) and Bioactivities (Antioxidant Activity and Total Phenolic and Flavonoid Contents)

Fresh tomato fruits were squeezed using gauze to obtain the fresh juice extract. The TSS (total soluble solid) contents in 200 µL of fresh juice extract were measured using a portable refractometer for sugar measurements (Hanna Instruments, Inc., Padova, Italy). The TA (titratable acidity) was determined using the formal titration method, as described previously by Suh et al. [40]. Each of the juice extracts (10 mL) was diluted with distilled water (40 mL), and the TA was estimated by titrating it using 0.1 N NaOH solution to the pH end-point of 8.4.

The DPPH assay was performed as described previously by Suh et al. with slight modifications. Briefly, 20 µL of tomato leaf, root, and fruit sample extracts were mixed with 180 µM of 0.2 mM DPPH ethanol solution in a 96-well plate for 20 min at room temperature. The resulting sample absorbance was recorded using a spectrophotometer at 515 nm, and the results were expressed as the Trolox equivalent antioxidant capacity (mM).

Total phenolic content (TPC) and total flavonoid content (TFC) were determined as described previously by Suh et al. [40] To evaluate TPC, 20 µL of sample extracts were mixed with 100 µL of 0.2 N Folin-Ciocalteu’s phenol reagent in a 96-well plate, and the reaction was incubated at room temperature in the dark. After incubation for 5 min, 80 µL of 7.5% NaCO_3_ were added to the mixture, which was then incubated for 60 min at room temperature. Finally, the absorbance was measured using a spectrophotometer at 750 nm. The results were expressed as gallic acid equivalents (ppm).

To measure TFC, 20 µL of sample extracts were added to 180 µL of 90% diethylene glycol and 20 µL of 1 N NaOH solution, and then incubated for 60 min at room temperature in the dark. The absorbance was measured at 405 nm, and the results were presented as the naringin equivalents (ppm).

### 4.7. Data Processing and Multivariate Statistical Analysis

The GC-TOF-MS raw data files were converted to NetCDF (*.cdf) using the LECO Chroma TOF software (version 4.44). The UHPLC-LTQ-Orbitrap-MS raw data files were converted to NetCDF (*.cdf) format using Thermo Xcalibur software (version 2.1, Thermo Fisher Scientific). Converted CDF data were preprocessed with the MetAlign software package (http://www.metalign.nl) for peak detection, retention time correction, and alignment. The resulting data were exported to an Excel file. Data sets then were normalized to internal standards based on peak intensities of daidzein (both in GC-TOF-MS and UHPLC-LTQ-Orbitrap-MS) for analytical quality of the results. Statistical analysis was performed using SIMCA-P+ software (version 12.0, Umetrics, Umea, Sweden) to compare metabolites among samples. Principal component analysis (PCA) and partial least squares discriminant analysis (PLS-DA) modeling were performed to compare the different metabolites of the samples.

The discriminant metabolites were selected based on VIP values > 0.7 and tested for significance at *p*-value < 0.05. The selected metabolites were tentatively identified by comparison with various data, including mass fragment patterns, retention time, and mass spectrum of analysis data for standard compounds under same conditions published papers and commercial databases, such as the National Institutes of Standards and Technology (NIST) Library (version 2.0, 2011, FairCom, Gaithersburg, MD, USA), The Dictionary of Natural Products (version 16:2, 2007, Chapman and Hall, USA), Wiley 8, BioCyc Database Collection (https://biocyc.org/), and the Human Metabolome Database (HMDB; http://www.hmdb.ca/). Significance (*p* < 0.05) were tested by one-way ANOVA using Statistica (version 7.0, StatSoft Inc., Tulsa, OK, USA). A heat map was visualized using MEV software.

## 5. Conclusions

In conclusion, the untargeted metabolomic analysis of tomato revealed that Mg oversupply leads to altered root metabolomes, which can be attributed to the stress response of tomato plants to high magnesium levels. In terms of tomato plant metabolism, the present study demonstrated that differential concentrations of metabolites alter the commercially desirable traits of tomato fruits. This is particularly apparent in the root of the plant, which is the first organ to encounter Mg stress. We suggested that tomatoes allocate metabolite resources to roots through activating the metabolism of carbohydrates and polyamines. On the contrary, the metabolism of carbohydrates, amino acids, polyamines, phenylpropanoid was downregulated in leaves. In addition to these findings, Mg oversupply induced marked changes in the fruit metabolome. This was particularly apparent in the primary metabolites, which adversely affect its organoleptic properties and quality-determining traits. Thus, the present study concludes that the excess accumulation of Mg in the soil can have detrimental effects on the overall plant development and tomato fruit quality.

## Supplementary Materials

The following are available online at https://www.mdpi.com/2218-1989/9/10/231/s1, Table S1: Differential secondary metabolites identified by UHPLC-LTQ-Orbitrap-MS in tomato leaf, root, and fruit samples cultivated under Mg oversupply. Table S2: Differential primary metabolites identified by GC-TOF-MS in tomato leaf, root, and fruit samples cultivated under Mg oversupply. Figure S1: Principle component analysis (PCA) score plots for metabolites in tomato leaves, roots, and fruits under control and Mg oversupply conditions based on the GC-TOF-MS and UHPLC-Orbitrap-MS data set. (a) Score plot for control (●), MgH (●), and MgEH (●) leaf samples, (b) score plot for control (♦), MgH (♦), and MgEH (♦) root samples, (c) score plot for control (▲), MgH (▲), and MgEH (▲) fruit samples. Figure S2: Heat map representation for the relative abundance of significantly discriminant metabolites (VIP > 0.7) based on the root and leaf PLS-DA model (Figure 2a,b). The values represent the fold change with respect to control. ^a^ indicates significant differences (*p*-value < 0.05) between control and each treatment. Figure S3: Correlation map between the fruit metabolite levels and observed FFW, TSS, and TA. Each metabolite is identified as significantly different metabolites through PLS-DA (Figure 2c). Each square indicates Pearson’s correlation coefficient of a pair of metabolites and assayed activities. The red color indicates a positive (0 < r <1) correlation and the blue colors indicate negative (−1 < r < 0) correlation. Figure S4: Results of bioactivities, including DPPH, TFC, and TPC in tomato leaf (a) and root (b) induced by Mg oversupply. Different letters in the bar graph indicate significant difference by ANOVA followed by Duncan’s multiple-range test (*p* value < 0.05).
