# Supplementary material for: Comparative Metabolomics Unravel the Effect of Magnesium Oversupply on Tomato Fruit Quality and Associated Plant Metabolism
Source: Metabolites. 2019 Oct 16;9(10):231. doi: 10.3390/metabo9100231 (PMC6835971; doi:10.3390/metabo9100231)
Supplement: Supplementary file 1 [file metabolites-09-00231-s001.zip › metabolites-614595-SI.docx]

**Table S1.** Differential secondary metabolites identified by UHPLC-LTQ-Orbitrap-MS in tomato leaves, roots, and fruit samples cultivated under Mg oversupply.

| **No.** | **RT^a^ (min)** | **Tentative Metabolite^b^** | **UHPLC-LTQ-Orbitrap-MS** | | | | | | | **References** |
| --- | --- | --- | --- | --- | --- | --- | --- | --- | --- | --- |
|  |  |  | **[M-H]^-^** | **[M+H]^+^** | **M.W.^c^** | **M.F.^d^** | **Delta ppm** | **MS^n^ fragments** | |  |
| ***Polyamines*** | | | | | | | | | |  |
| 1 | 1.27 | Caffeoyl putrescine | 249.1262 | 251.1376 | 250 | C13H18N2O3 | -1.428 |  | 249.1262>207.1223>135.0354 | Ref[S1] |
| 2 | 3.51 | Feruloyl putrescine | 263.1397 | 265.1541 | 264 | C14H20N2O3 | -1.542 |  | 265.1397>177.2327>144.9456 | Ref[S2] |
| 3 | 4.06 | Feruloyl agmatine | 305.1614 | 307.1763 | 306 | C15H22N4O3 | -0.766 |  | 307.1763>290.3241>247.1610>177.0435 | Ref[S2] |
| 4 | 4.56 | Tris(dihydrocaffeoyl)spermine | 693.3488 | 695.3649 | 694 | C37H50N4O9 | -2.340 |  | 693.3488>571.4763>529.5952>407.3401 | Ref[S1] |
| 5 | 4.93 | Feruloyl octopamine | 328.1224 | 330.1322 | 329 | C18H19NO5 | -1.847 |  | 328.1224>310.3226>295.3177>161.0563 | Ref[S1] |
| 6 | 5.59 | Coumaroyl tyramine | 282.1130 | 284.1280 | 283 | C17H17NO3 | -2.080 |  | 284.1280>147.2196>118.9864 | Ref[S2] |
| 7 | 5.71 | Feruloyl tyramine | 312.1237 | 314.1385 | 313 | C18H19NO4 | -1.222 |  | 314.1385>177.2159>145.0326 | Ref[S2] |
| ***Phenylpropanoids*** | | | | | | | | | | |
| 8 | 3.7 | Caffeoylquinic acid^*^ | 353.0871 | 355.1024 | 354 | C16H18O9 | -0.185 |  | 353.0871>191.3054>173.0765 | Ref[S3] |
| 9 | 5.4 | Tricaffeoylquinic acid | 677.2859 | 679.2936 | 678 | C34H30O15 | -2.800 |  | 677.2859>515.3137>497.1244>353.0781 | Ref[S4] |
| 10 | 4.64 | Kaempferol rutinoside pentoside | 725.1963 | 727.2059 | 726 | C32H38O19 | -2.816 |  | 725.1963>593.1998>575.3190>327.0947 | Ref[S4] |
| 11 | 4.89 | Kaempferol rutinoside | 593.1545 | 595.4005 | 594 | C27H30O15 | -2.888 |  | 593.1545>285.1878>267.0215>257.0482 | Ref[S4] |
| 12 | 4.47 | Quercetin rutinoside pentoside | 741.1920 | 765.2547^e^ | 742 | C32H38O20 | -1.911 |  | 741.1920>723.2255>609.0939>300.3351 | Ref[S4] |
| 13 | 5.11 | Quercetin–glucose–rhamnose– apiose-ferulic acid | 917.2339 | 919.2522 | 918 | C42H46O23 | -3.915 |  | 917.2339>741.3862>723.2241>609.2237 | Ref[S5] |
| ***Steroidal saponins*** | | | | | | | | | | |
| 14 | 4.81 | Esculeoside A | 1268.5997 | 1270.5981 | 1269.5 | C58H95NO29 | -1.867 |  | 1270.5287>1210.7856>1048.6085>592.5248 | Ref[S6] |
| 15 | 4.85 | Hydroxytomatine | 1048.5378 | 1050.5411 | 1049.5 | C50H83NO22 | 0.258 |  | 1048.5378>916.6991>887.6090>754.6767 | Ref[S7] |
| 16 | 5.3 | Dehydrotomatine | 1030.5261 | 1032.5310 | 1031.5 | C50H81NO21 | 0.092 |  | 1030.5261>898.7277>869.5759>736.8016 | Ref[S8] |
| 17 | 5.41 | Tomatoside A | 1081.5474 | 1105.5339^e^ | 1082.5 | C51H86O24 | 2.888 |  | 1065.5421>903.4989>579.3245>517.3478 | Ref[S8] |
| 18 | 5.49 | Alpha-tomatine | 1032.5424 | 1034.5461 | 1033.5 | C50H83NO21 | 0.537 |  | 1032.5424>900.6953>870.7097>738.7258 | Ref[S8] |
| ***Lipids*** | | | | | | | | | | |
| 19 | 6.22 | 9,12,13-TriHODE | 327.2170 | 351.2148^e^ | 328 | C18H32O5 | -2.101 |  | 327.2170>309.2128>291.1649>171.0548 | Ref[S9] |
| 20 | 6.5 | 9,10,13-TriHOME | 329.2323 | 353.2303^e^ | 330 | C18H34O5 | -3.090 |  | 329.2323>311.2377>201.1186>171.1057 | Ref[S9] |
| 21 | 7.88 | LysoPC(18:3) | 562.3201^f^ | 518.3214 | 517 | C26H48NO7P | -4.037 |  | 562.3201>502.4997>277.2433 | Ref[S10] |
| 22 | 8.22 | LysoPE(18:2) | 476.2824 | 478.2929 | 477 | C23H44NO7P | -3.434 |  | 478.2929>460.3062>337.4749 | Ref[S10] |
| 23 | 8.27 | LysoPC(18:2) | 564.3337^f^ | 520.3399 | 519 | C26H50NO7P | -3.714 |  | 520.3399>502.3292>184.0460 | Ref[S10] |
| 24 | 8.39 | LysoPA(18:3) | 431.2247 | 433.2328 | 432 | C21H37O7P | -1.284 |  | 431.2247>152.9150 | Ref[S11] |
| 25 | 8.41 | PI(16:0) | 571.2917 | 573.3004 | 572 | C25H49O12P | -4.388 |  | 571.2917>391.2625>315.0678>255.2483 | Ref[S12] |
| 26 | 8.6 | LysoPE(16:0) | 452.2807 | 454.2906 | 453 | C21H44NO7P | -4.118 |  | 454.2906>436.4770>393.2216>313.5041 | Ref[S10] |
| 27 | 8.68 | LysoPC(16:0) | 540.3342^f^ | 496.3370 | 495 | C24H50NO7 P | -1.805 |  | 496.3370>478.5829>419.2864>184.0415 | Ref[S10] |
| 28 | 8.77 | LysoPC(18:1) | 566.3478^f^ | 522.3525 | 521 | C26H52NO7P | -2.002 |  | 522.3525>504.4971>184.0195 | Ref[S10] |
| 29 | 8.91 | LysoPA(18:2) | 433.2384 | 435.2484 | 434 | C21H39O7P | -1.485 |  | 433.2384>152.9257 | Ref[S11] |
| 30 | 9.45 | LysoPA(16:0) | 409.2382 | 411.0600 | 410 | C19H39O7P | 6.028 |  | 409.2382>152.9535 | Ref[S11] |
| 31 | 9.52 | LysoPC(18:0) | 568.3663^f^ | 524.3712 | 523 | C26H54O7NP | -1.575 |  | 524.3712>506.5915>184.0182 | Ref[S10] |

^a^ Retention time; ^b^ Metabolites selected by VIP value > 0.7 based on PLS-DA (Figure 2); ^c^ Molecular weight; ^d^ Molecular formula; ^e^ [M+NA]^+^; ^f^ [M+FA-H]^-^; * Caffeoylqunic acid is one of the 3-O-caffeoylqunic acid, 4-O-caffeoylqunic acid and 5-O-caffeoylqunic acid.

**Table S2.** Differential primary metabolites identified by GC-TOF-MS in tomato leaves, roots, and fruit samples cultivated under Mg oversupply.

| **NO.** | **Tentative identification ^a^** | **GC-TOF-MS** | | | | |
| --- | --- | --- | --- | --- | --- | --- |
|  |  | **RT(min) ^b^** | **Identified**  **ion (m/z) ^c^** | **Mass Fragment** | **TMS ^d^** | **ID ^e^** |
| *Carbohydrates* | | | | |  |  |
| 1 | Threonic acid | 9.67 | 292 | 117 75 55 102 | 4 | MS |
| 2 | Xylose | 10.61 | 103 | 103 217 117 74 | 4 | STD |
| 3 | Fucose | 10.92 | 117 | 117 160 118 133 | 4 | STD |
| 4 | Fructose | 12.07 | 307 | 103 217 74 133 | 5 | STD |
| 5 | Glucose | 12.36 | 160 | 160 205 103 74 | 5 | STD |
| 6 | Gluconic acid | 12.91 | 333 | 103 74 333 217 | 5 | STD |
| 7 | *myo*-Inositol | 13.47 | 217 | 217 191 318 129 | 6 | STD |
| 8 | Sucrose | 16.56 | 437 | 129 169 103 217 | 8 | STD |
| 9 | Maltose | 16.99 | 361 | 204 103 75 129 | 8 | STD |
| *Organic acids* | |  |  |  |  |  |
| 10 | Lactic acid | 4.82 | 117 | 117 75 66 59 | 2 | STD |
| 11 | Benzoic acid | 6.81 | 179 | 105 77 179 135 | 1 | MS |
| 12 | Succinic acid | 7.44 | 247 | 75 55 56 148 | 2 | STD |
| 13 | Propanoic acid | 7.64 | 189 | 189 102 103 133 | 3 | MS |
| 14 | Fumaric acid | 7.72 | 245 | 245 75 143 133 | 2 | STD |
| 15 | Malic acid | 9.05 | 245 | 133 148 101 233 | 3 | STD |
| 16 | Citric acid | 11.63 | 273 | 273 75 67 74 | 4 | STD |
| 17 | Oxoglutaric acid | 11.85 | 173 | 55 173 157 75 | 2 | STD |
| *Amino acids* | |  |  |  |  |  |
| 18 | Alanine | 5.27 | 116 | 116 75 103 117 | 2 | STD |
| 19 | Valine | 6.51 | 144 | 144 100 145 218 | 2 | STD |
| 20 | Isoleucine | 7.28 | 158 | 158 57 75 59 | 2 | STD |
| 21 | Glycine | 7.41 | 174 | 174 100 175 117 | 3 | STD |
| 22 | Serine | 7.91 | 188 | 204 100 218 75 | 3 | STD |
| 23 | Threonine | 8.16 | 101 | 101 117 59 74 89 | 3 | STD |
| 24 | GABA | 9.40 | 174 | 174 86 100 304 | 3 | STD |
| 25 | Glutamic acid | 10.08 | 246 | 246 75 128 84 | 3 | STD |
| 26 | Phenylalanine | 10.18 | 218 | 218 192 100 75 | 2 | STD |
| 27 | Asparagine | 10.50 | 116 | 116 132 75 74 | 3 | STD |
| *Fatty acids* | |  |  |  |  |  |
| 28 | Palmitic acid |  |  |  |  |  |
| 29 | Linoleic acid | 14.00 | 337 | 75 67 81 55 | 1 | STD |
| 30 | Stearic acid | 14.16 | 341 | 117 75 132 129 | 1 | STD |
| 31 | Oleamide | 15.15 | 338 | 75 131 144 116 | 1 | STD |
| *Others* | |  |  |  |  |  |
| 32 | Quinic acid | 11.94 | 255 | 345 255 346 191 | 5 | STD |
| 33 | Adenosine diphosphate | 7.11 | 314 | 299 133 300 74 | 3 | STD |

^a^ Metabolites selected by VIP value > 0.7 based on each PLS-DA model (Figure 2); ^b^ Retention time; ^c^ The selected ion is m/z value for identification and quantification; ^d^ TMS : the number of trimethylsilyl groups; ^e^ Identification. MS, mass spectrum was confirmed with the National Institutes of Standards and Technology (NIST) database and in-house libraries; STD, mass spectrum was consistent with that of the standard compounds.


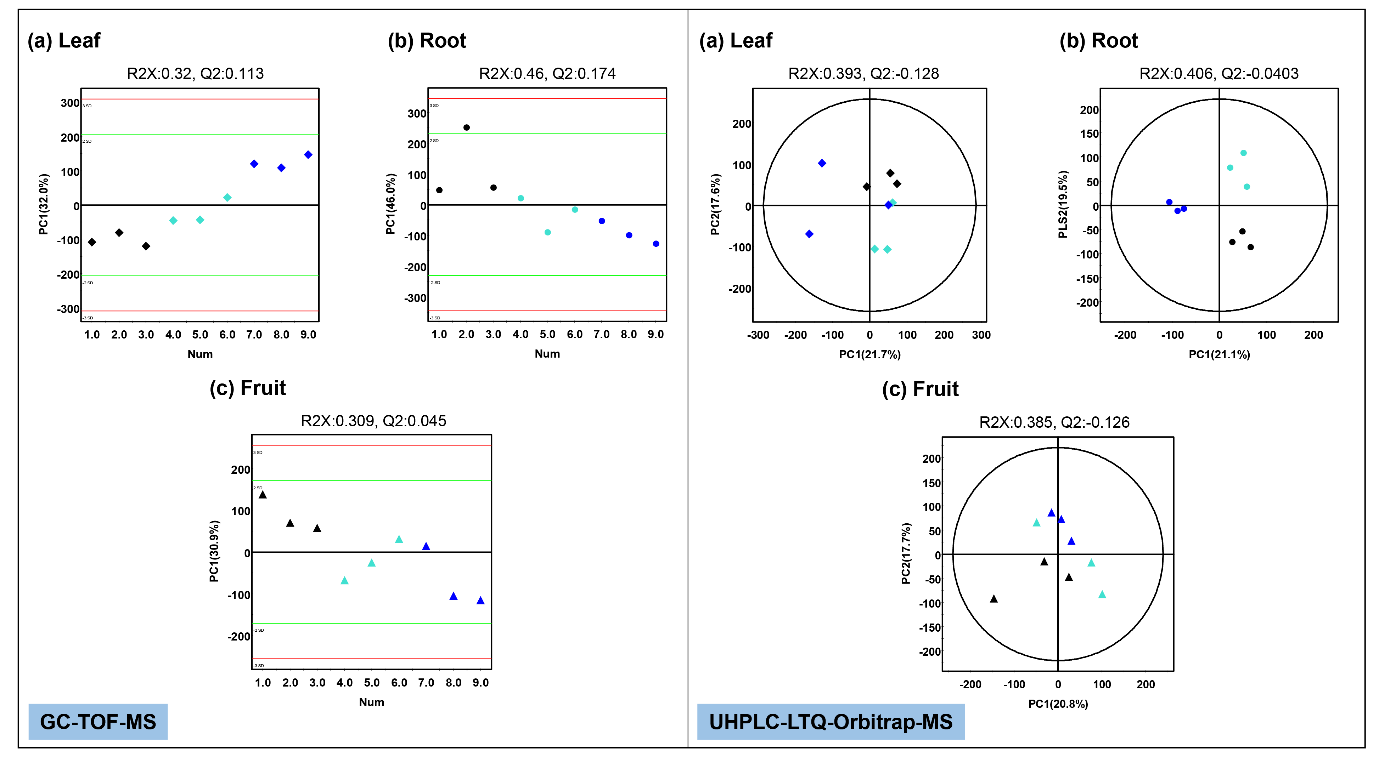


**Figure S1.** Principle component analysis (PCA) score plots for metabolites in tomato leaves, roots, and fruits under control and Mg oversupply conditions based on the GC-TOF-MS and UHPLC-Orbitrap-MS data set. a) score plot for control (●), MgH (●), and MgEH (●) leaf samples, b) score plot for control (◆), MgH (◆), and MgEH (◆) root samples, c) score plot for control (▲), MgH ▲), and MgEH (▲) fruit samples.


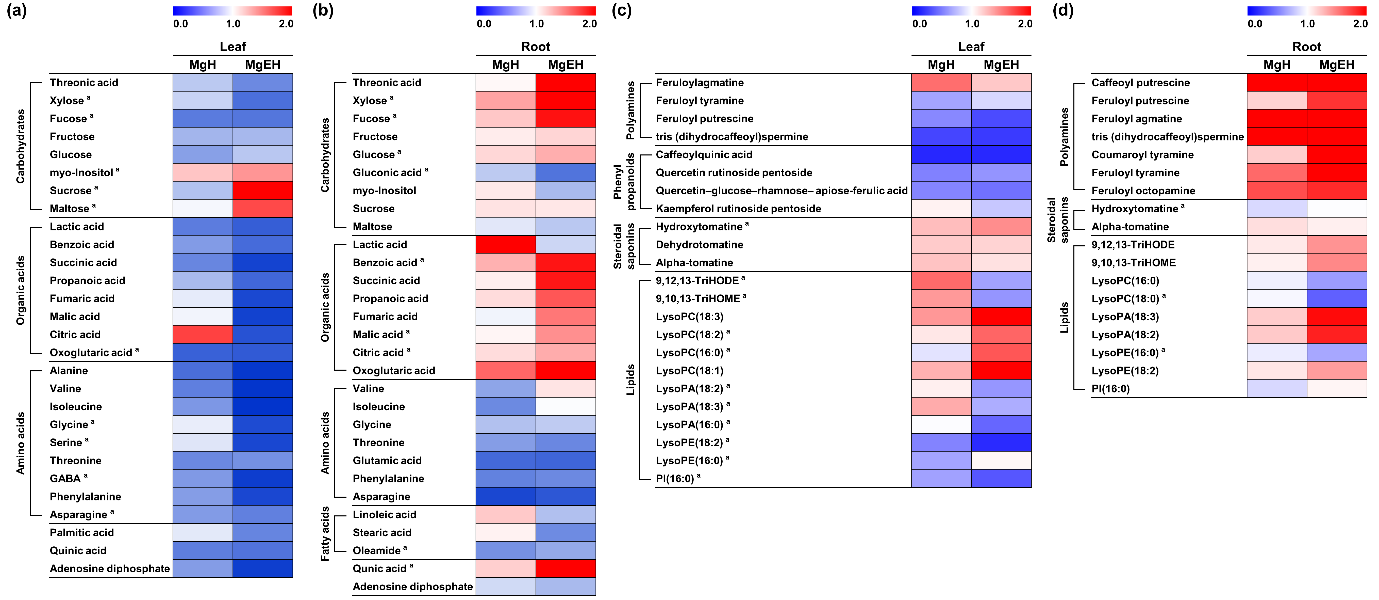


**Figure S2.** Heat map representation for the relative abundance of significantly discriminant metabolites (VIP > 0.7) based on the root and leaf PLS-DA model (Figure 2a,b). The values represent the fold change with respect to control. ^a^ indicate significant differences (p-value < 0.05) between control and each treatment.

**
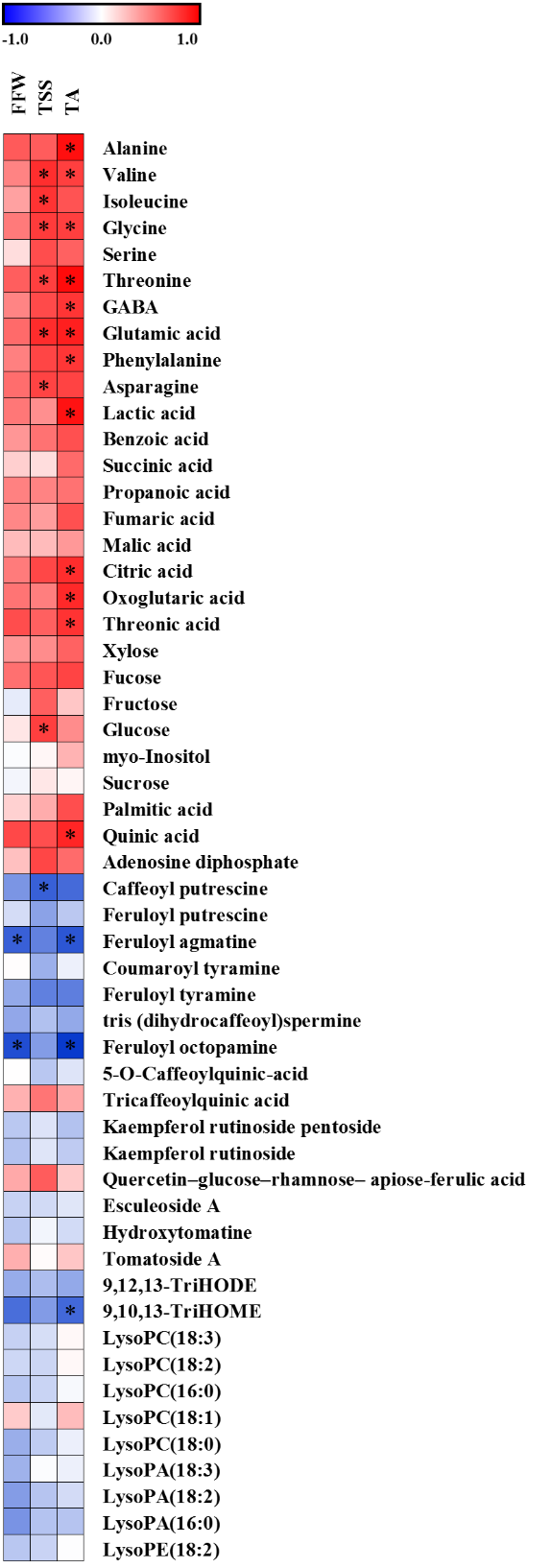
**

**Figure S3.** Correlation map between the fruit metabolite levels and observed FFW, TSS, and TA. Each metabolite is identified as a significantly different metabolites through PLS-DA (Figure 2c). Each square indicates Pearson’s correlation coefficient of a pair of metabolites and assayed activities. The red color indicates a positive (0 < r <1) correlation and the blue colors indicate negative (-1 < r < 0) correlation.

**
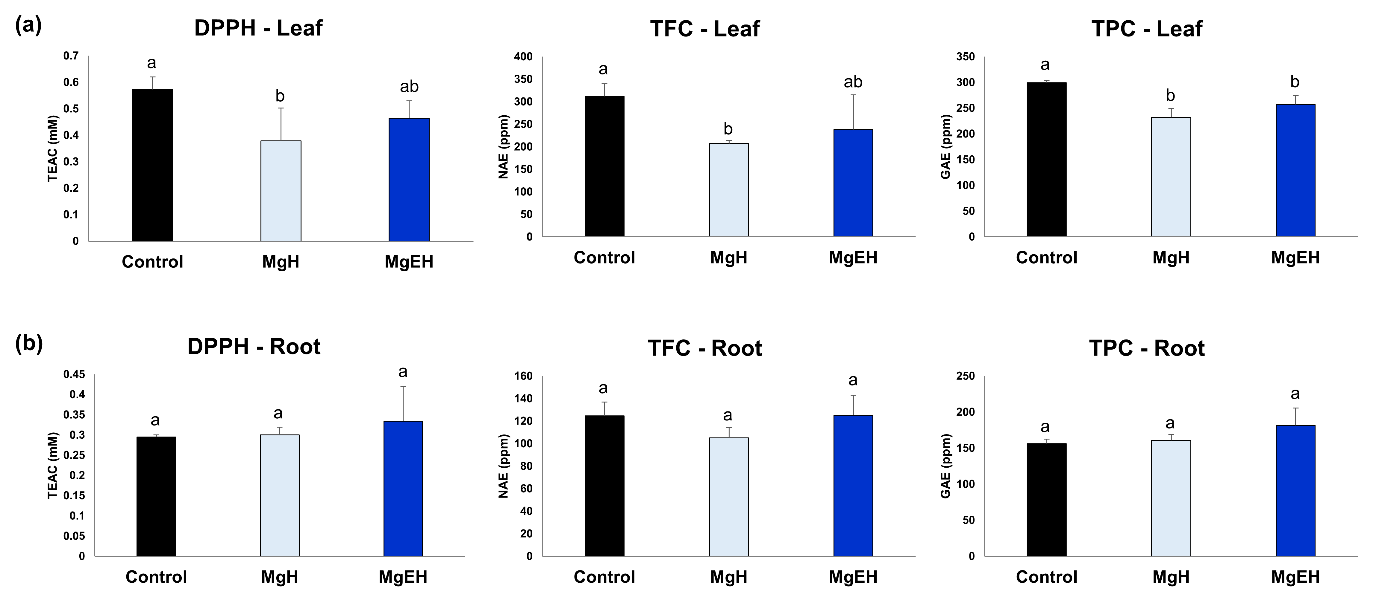
**

**Figure S4.** Results of bioactivities including DPPH, TFC, and TPC in tomato leaves (a) and roots (b) induced by Mg oversupply. Different letters in the bar graph indicate significant difference by ANOVA followed by Duncan’s multiple-range test (p value < 0.05).

**Supplementary references**

1. Narváez-Cuenca, C. E.; Vincken, J. P.; Gruppen, H. Identification and quantification of (dihydro) hydroxycinnamic acids and their conjugates in potato by UHPLC–DAD–ESI-MS^n^. *Food Chem.* **2012**, *130*(3), 730-738.
2. Voynikov, Y.; Zheleva-Dimitrova, D.; Gevrenova, R.; Lozanov, V.; Zaharieva, M. M.; Tsvetkova, I.; Najdenski, H.; Yagi, S.; Almoulah, N. F.; Momekov, G. Hydroxycinnamic acid amide profile of *Solanum schimperianum* Hochst by UPLC-HRMS. *Int. J. Mass Spectrom*. **2016**, *408*, 42-50.
3. Ricci, A.; Cirlini, M.; Calani, L.; Bernini, V.; Neviani, E.; Del Rio, D.; Galaverna, G.; Lazzi, C. In vitro metabolism of elderberry juice polyphenols by lactic acid bacteria. *Food Chem.* **2019**, *276*, 692-699.
4. Anton, D.; Bender, I.; Kaart, T.; Roasto, M.; Heinonen, M.; Luik, A.; Püssa, T. Changes in polyphenols contents and antioxidant capacities of organically and conventionally cultivated tomato (*Solanum lycopersicum* L.) fruits during ripening*. Int. J. Anal. Chem.* **2017**, *2017*
5. Chanforan, C.; Loonis, M.; Mora, N.; Caris-Veyrat, C.; Dufour, C. The impact of industrial processing on health-beneficial tomato microconstituents. *Food Chem.* **2012**, *134(4)*, 1786-1795.
6. Iijima, Y.; Fujiwara, Y.; Tokita, T.; Ikeda, T.; Nohara, T.; Aoki, K.; Shibata, D. Involvement of ethylene in the accumulation of esculeoside A during fruit ripening of tomato (*Solanum lycopersicum*). *J. Agric. Food Chem.* **2009**, *57(8)*, 3247-3252.
7. Roldan, M. V. G.; Engel, B.; de Vos, R. C.; Vereijken, P.; Astola, L.; Groenenboom, M.; Geest, H. V. D.; Bovy, A.; Molenaar, J.; Eeuwijk, F. V.; Hall, R. D. Metabolomics reveals organ-specific metabolic rearrangements during early tomato seedling development. *Metabolomics* **2014**, *10(5)*, 958-974.
8. Gómez-Romero, M.; Segura-Carretero, A.; Fernández-Gutiérrez, A. Metabolite profiling and quantification of phenolic compounds in methanol extracts of tomato fruit. *Phytochemistry* **2010**, *71(16)*, 1848-1864.s
9. Son, S. Y.; Lee, S.; Singh, D.; Lee, N. R.; Lee, D. Y.; Lee, C. H. Comprehensive secondary metabolite profiling toward delineating the solid and submerged-state fermentation of *Aspergillus oryzae* KCCM 12698. *Front. Microbiol.* **2018**, *9*, 1076.
10. Wei, Z.; Xi, J.; Gao, S.; You, X.; Li, N.; Cao, Y.; Dong, X. Metabolomics coupled with pathway analysis characterizes metabolic changes in response to BDE-3 induced reproductive toxicity in mice. *Sci. Rep.* **2018**, *8(1)*, 5423.
11. Benesch, M. G.; Tang, X.; Maeda, T.; Ohhata, A.; Zhao, Y. Y.; Kok, B. P.; Dewald, J.; Hitt, M.; Curtis, J. M.; Mcmullen, T. P.; Brindley, D. N. Inhibition of autotaxin delays breast tumor growth and lung metastasis in mice. *FASEB J.* **2014**, *28(6)*, 2655-2666.
12. Wu, H.; Chen, Y.; Li, Q.; Gao, Y.; Zhang, X.; Tong, J.; Zhang, Z.; Hu, J.; Wang, D.; Zeng, S.; Li, Z. Intervention effect of Qi-Yu-San-Long Decoction on *Lewis* lung carcinoma in C57BL/6 mice: Insights from UPLC–QTOF/MS-based metabolic profiling. *J. Chromatogr. B* **2018**, *1102*, 23-33.
